# On the analyses of graphene oxide/polypyrrole/zinc oxide nanocomposites

**DOI:** 10.1038/s41598-025-20194-4

**Published:** 2025-10-01

**Authors:** Asmaa Ibrahim, Hanan Elhaes, Nada A. Khaled, Medhat A. Ibrahim

**Affiliations:** 1 Physics Department, Faculty of Women for Arts, Science and Education, Ain Shams University, Cairo, 11757 Egypt; 2 Therapeutic Chemistry Department, National Research Centre, 33 El-Bohouth St., Dokki, Giza, 12622 Egypt; 3 Spectroscopy Department, National Research Centre, 33 El-Bohouth St., 12622, Dokki, Giza, Egypt; 4 Molecular Modeling and Spectroscopy Laboratory, Centre of Excellence for Advanced Science, National Research Centre, 33 El-Bohouth St., 12622, Dokki, Giza, Egypt

**Keywords:** Graphene oxide, Polypyrrole, ZnO, DFT and alanine, Chemistry, Materials science, Nanoscience and technology

## Abstract

Graphene oxide/Polypyrrole/Zinc oxide GrO/PPy/ZnO nanocomposite was investigated for possible interaction with alanine using B3LYP/LANL2DZ model. Results indicated that GrO/PPy/ZnO exhibited notable electronic accessibility with a total dipole moment (TDM) of 5.62 Debye and HOMO-LUMO energy gap of 1.64 eV, which was significantly modulated upon alanine binding. COOH functionalization induced the greatest reduction in ionization potential (from 3.03 eV to 2.56 eV) alongside increased electron affinity (4.68 to 4.77 eV), while NH₂ functionalization showed moderate improvements (ionization potential to 2.67 eV, electron affinity to 4.75 eV). Quantum Theory of Atoms in Molecules (QTAIM) analysis revealed distinct binding characteristics: NH₂-bound systems formed multiple Zn–N and Zn–O coordination bonds with flexible interaction networks, while COOH-bound systems exhibited fewer but stronger, more localized coordination and hydrogen bonds. Molecular electrostatic potential (MESP) demonstrated enhanced positive potential near NH₂ binding sites and pronounced dipolar features around COOH regions. Non-covalent interaction (NCI) and reduced density gradient (RDG) analyses revealed that COOH functionalization produced more concentrated blue domains, indicating stronger interactions and enhanced selectivity. Density of states (DOS) showed notable band gap reduction after composite formation and functionalization, with GrO/PPy/ZnO exhibiting the most favorable electronic structure for charge transport. Alanine binding lowered system polarity (TDM: 2.81 Debye for COOH and 2.77 D for NH₂) while preserving structural stability, as shown by slight changes in chemical hardness. Overall, COOH-functionalized GrO/PPy/ZnO shows the best balance of reactivity, stability, and selective binding, with favorable electrostatics and strong interactions, highlighting its promise as an efficient amino acid sensor.

## Introduction

While most polymers are typically insulators, some are considered conducting polymers, which are comparable in their properties to those of inorganic semiconductors and metals^1,2^. Those classes of conducting polymers show single and double bonds in the conjugated carbon chain, which create highly nonlocal, polarized, electron-dense π bonds responsible for their unique electrical and optical properties^3^. Polypyrrole (PPy), among other conducting polymers show stable structure, conductive easy forming composites, beside electrochemical activity, which dedicate it for sensing applications^4,5^. The deep need for sensors operating at room temperature derive the root toward conducting polymers^6^. In this sense, polymer composites, especially nanocomposite could be the solution for room temperature sensors. These polymers show interesting electronic as well as electrochemical properties^7^. Such class of composites is promising based on their enhanced optical, electronic and mechanical properties^8^. Graphene oxide GrO is a 2D hexagonal lattice carbon material that finds ways to different applications owing to its optical and electronic properties^9^. The promising applications of such carbon-based material are due to their high charge carrier mobility, and mechanical qualities^10^. Graphene oxide enhances the optical and band gap energy of PPy as they form composite together^11^.

As stated earlier that the PPys π-electron-rich endows it with a pronounced affinity for specific metal ions, facilitating interactions characterized by electrostatic attraction and coordination. The inherent conductivity of PPy is further harnessed to enhance its capabilities in electrochemical sensing applications^12,13^. The functionalization of polymers involves modifying their chemical structures to increase properties such as solubility, adhesion, and reactivity, which is necessary to tailor their properties to specific applications, improve their performance, and extend their usability^14,15^. Amino-functionalized graphene oxide/polypyrrole (AM-GrO/PPy) composite-based novel sensing platform was established to monitor lead ions (Pb^2+^) at high sensitivity^16^. It was stated that, the AM-GrO/PPy composite emerges as efficient sensor acts for the electrochemical detection of Pb^2+^, holding significant potential for environmental monitoring and the protection of human health. The PPy-GrO composite is also presented as a promising material and a sensor device developed using interdigitated copper electrode on copper clad is a cost-effective approach for detection of CO^17^. Development of cost effective and selective gas sensor is a hot topic of research^18^. Molecular modeling is a class of computational work elucidating the electronic, physical and chemical properties of a wide range of molecular systems^19,20^.

Molecular modeling was used to study PLA/GrO/ZnO and PLA/GrO/Cu_2_O interacting with gases and volatile organic compounds. Results indicated that these composites could be used as gas sensors^21^. A study based on computational molecular modeling indicated that the graphene oxide/WO_3_/polyvinylidene fluoride composite could be applied as biosensor^22^. It is stated that molecular modeling introduced important computational data that supports the experimental findings. This data was able to describe the mechanism of interaction between the nanocomposite surface and the studied gases^23–25^.

DFT has been used to study the interaction of cysteine with boron nitride nanotubes, which could lead to the development of new nano sensors and nano carriers for this and other amino acids^26^. In biomedical applications, DFT has also been used to identify hybrid B12N12/ZIF-8 nanoclusters as a potential platform for sensing the drug AMP. A detailed DFT analysis of their adsorption behavior, electronic properties, and intermolecular interactions showed their potential to effectively bind to and detect the drug^27^. Furthermore, DFT allows researchers to track key parameters like QTAIM and NCI to study the adsorption of organic structures on nanomaterials^28^.

In this work, a ternary nanocomposite comprising graphene oxide (GrO), polypyrrole (PPy), and zinc oxide (ZnO) is computationally designed and characterized to evaluate its potential for alanine sensing. The study focuses on elucidating how surface functionalization with amino (NH₂) and carboxyl (COOH) groups modulates the composite’s electronic and reactive properties. A comprehensive set of computational analyses is performed, including total dipole moment (TDM), HOMO–LUMO energy gap, Quantum Theory of Atoms in Molecules (QTAIM) analysis, molecular electrostatic potential (MESP) mapping, and global reactivity descriptors. Additionally, Non-Covalent Interaction (NCI) and Reduced Density Gradient (RDG) analyses are employed to prove weak intermolecular interactions between alanine and the functionalized composites. Density Functional Theory (DFT) calculations are used to determine ionization potential, electron affinity, chemical hardness, absolute softness, electronic chemical potential, and electrophilicity index, enabling quantitative assessment of changes upon alanine binding. The ultimate objective is to compare the sensing performance of NH₂- and COOH-functionalized systems, establish correlations between electronic structure modulation and selective analyte recognition, and identify the functionalization strategy that achieves the optimal balance of sensitivity, selectivity, and structural stability for high-performance amino acid detection.

## Computational details

The model system investigated in this study comprises alanine, graphene oxide (GrO), polypyrrole (PPy), and zinc oxide (ZnO).

**Fig. 1:**
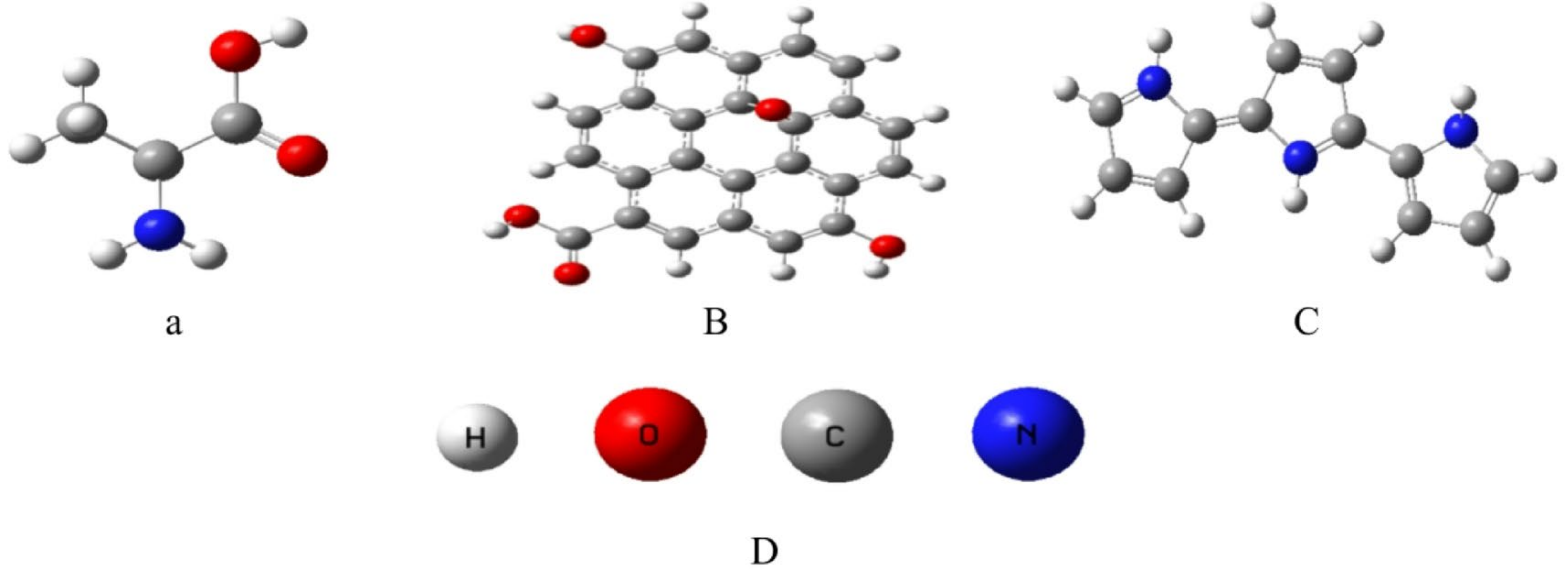
Optimized model structures of the individual components used in the study: (**a**) Alanine; (**b**) Graphene oxide (GrO); (**c**) Polypyrrole (PPy); (**d**) Labeled atomic sites for identification within the studied molecules.

Figure 1a shows the optimized structure of alanine, selected as a minimal amino acid model due to its two key functional groups, the amino group (NH₂) and the carboxyl group (COOH), which act as primary hydrogen-bonding sites and dominate amino acid–surface interactions. Alanine was chosen for three reasons: (i) computationally, it is the simplest chiral amino acid containing both NH₂ and COOH moieties, enabling clear mechanistic interpretation at reduced computational cost; (ii) biologically, its plasma concentration is a clinically relevant biomarker for liver function, metabolic disorders, and muscle health; and (iii) mechanistically, its interaction via amino and carboxyl groups is representative of most amino acids, thereby supporting generalization of the results to broader sensing applications. Importantly, these two universal functional groups are the principal determinants of amino acid binding to nanomaterials through hydrogen bonding, electrostatics, and coordination interactions. Hence, the interaction mechanisms observed in this study capture the fundamental binding motifs common across the amino acid family. This methodological approach has been widely adopted in both computational and experimental investigations, where alanine serves as a model probe for generalizing amino acid adsorption and sensing behavior on nanostructured surfaces^29^. While side-chain variations may introduce secondary effects (e.g., hydrophobic or aromatic interactions), the dominant NH₂/COOH-driven interactions captured by our alanine model provide a robust foundation for extrapolating the findings to other amino acids.

Figure 1b presents the GrO model, represented as a two-dimensional nanostructure functionalized with hydroxyl (OH) and carboxyl (COOH) groups. These oxygen-containing functionalities are distributed across the surface, enabling interactions through surface coordination rather than chain-length effects. Figure 1c shows the PPy model composed of three repeating pyrrole units, and Fig. 1d provides the atomic site labeling for reference.

**Fig. 2:**
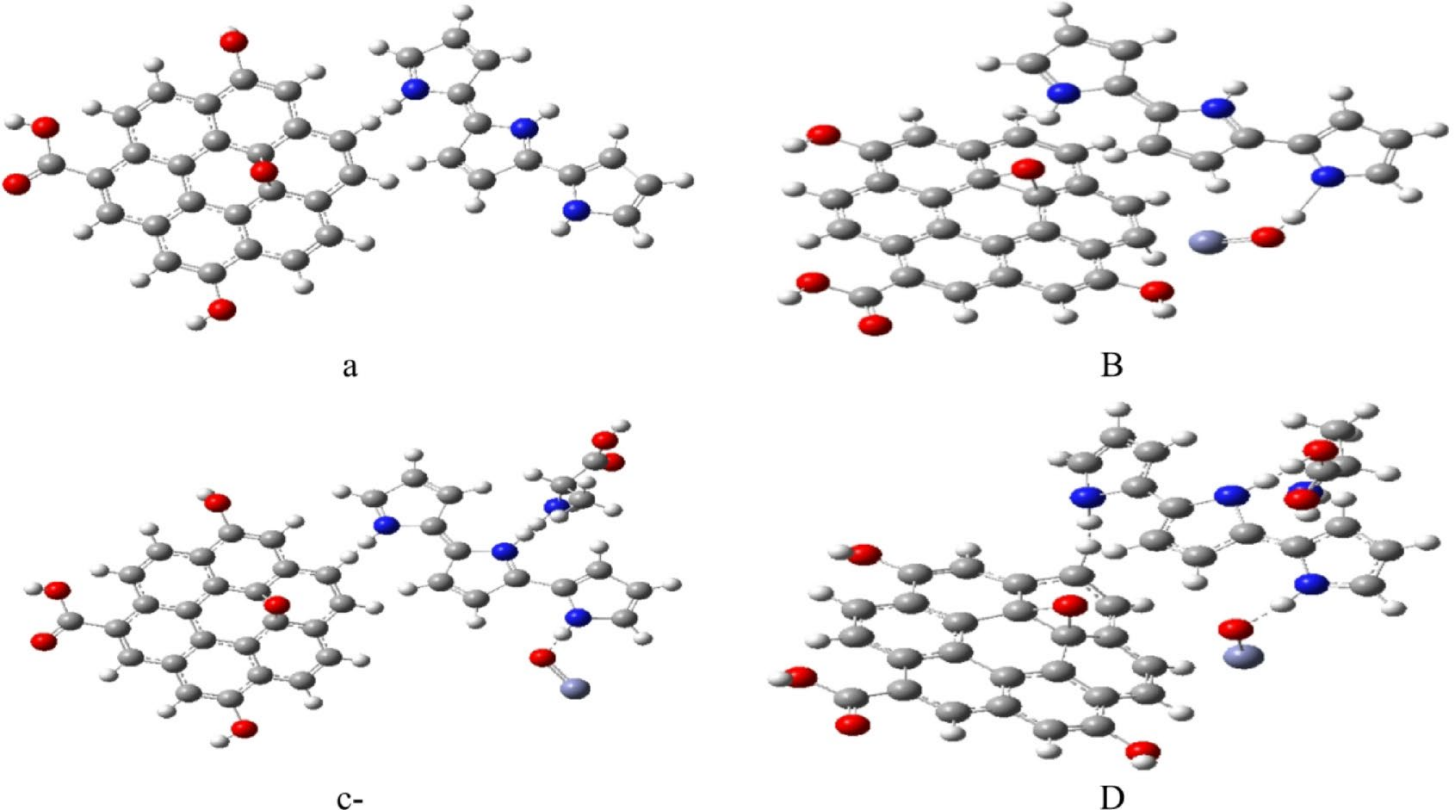
Optimized model structures of the composite and interaction systems: (**a**) GrO/PPy binary composite; (**b**) GrO/PPy/ZnO ternary nanocomposite; (**c**) GrO/PPy/ZnO/NH₂ complex interacting via the amino group of alanine; (**d**) GrO/PPy/ZnO/COOH complex interacting via the carboxyl group of alanine.

Figure 2a depicts the binary GrO/PPy composite, while Fig. 2b illustrates the ternary GrO/PPy/ZnO system obtained by incorporating ZnO nanoparticles. The choice of GrO, ZnO, and a three-unit PPy chain achieves a balance between computational tractability and accuracy. Comparable model sizes have been shown to capture essential physicochemical interactions and reproduce interfacial properties observed in larger systems, thereby enabling accurate predictions without prohibitive computational demands.

The interaction of alanine with the ternary composite was examined in two configurations: (i) through the amino group (**GrO/PPy/ZnO/NH₂**), as shown in Fig. 2c, and (ii) via the carboxyl group (**GrO/PPy/ZnO/COOH**), as shown in Fig. 2d.

All calculations were carried out using the Gaussian 09 (G09) software package^30^, running on a personal workstation at the Molecular Modeling and Spectroscopy Laboratory, Centre of Excellence for Advanced Science, National Research Centre, Egypt. Geometry optimizations were performed using density functional theory (DFT) at the B3LYP level of theory, employing Becke’s three parameter exchange functional combined with the Lee-Yang-Parr correlation functional^31–33^. The Los Alamos National Laboratory 2 Double-Zeta (LANL2DZ) basis set was applied, suitable for systems containing transition metals such as zinc^34^.

This choice ensured reliable treatment of Zn while maintaining computational feasibility for the full nanocomposite. Although no additional benchmarking with larger split-valence basis sets was performed for the organic components, LANL2DZ has been widely applied in similar hybrid systems, and the consistency of our descriptors (ΔE, TDM, QTAIM, NCI) supports the reliability of the obtained results. Benchmarking with mixed basis sets (e.g., 6-31G for light atoms) will be considered in future work to further refine accuracy. Optimizations proceeded until all convergence criteria were satisfied, including thresholds for the maximum force, root mean square (RMS) force, maximum displacement, and RMS displacement. The calculated change in total energy ranged from − 9.819654D-09 to -1.441622D-08 Hartree, indicating successful convergence and energy minimization of the model systems.

In addition to geometry optimization, a range of theoretical properties and molecular descriptors was calculated, including total dipole moment (TDM), HOMO–LUMO energy gap (ΔE), and global reactivity descriptors [ionization potential (I), electron affinity (A), chemical hardness (η), absolute softness (S), electronic chemical potential (µ), electrophilicity index (ω), and electronegativity (χ)]. Bond topology and hydrogen-bonding interactions were analyzed using Quantum Theory of Atoms in Molecules (QTAIM), while molecular electrostatic potential (MESP) mapping was employed to visualize charge distribution and reactive sites. Non-covalent interaction (NCI) and reduced density gradient (RDG) analyses were performed to characterize weak intermolecular forces such as hydrogen bonding, π–π stacking and van der Waals interactions. Density of states (DOS) calculations were also carried out to examine modifications in the electronic structure upon composite formation and alanine functionalization.

## Results and discussion

### Physical parameters

Density Functional Theory (DFT) calculations of physical parameters provide key insights into the electronic structure, polarity, and potential reactivity of nanocomposite materials. Among these descriptors, the total dipole moment (TDM) and the HOMO–LUMO energy gap (ΔE) are widely recognized as indicators of molecular reactivity and intermolecular interaction potential^35,36^. In general, an increase in TDM accompanied by a decrease in ΔE reflects greater polarity, enhanced electronic delocalization, and higher susceptibility to chemical interaction.

The calculated TDM and ΔE values for all studied systems are presented in Table 1. Pristine graphene oxide (GrO) and polypyrrole (PPy) exhibited relatively low dipole moments (1.99 D and 2.19 D, respectively) and moderate electronic gaps (2.70 eV for GrO and 4.26 eV for PPy). Upon formation of the GrO/PPy composite, the TDM increased substantially to 5.71 D, indicating enhanced polarity and the emergence of additional potential binding sites. This increase was accompanied by a sharp reduction in the energy gap to 1.36 eV, signifying increased electronic delocalization and potential reactivity.

Incorporating ZnO into the composite produced only a slight decrease in TDM (5.62 Debye) and a modest increase in ΔE (1.64 eV), suggesting that ZnO slightly moderates the polarity without significantly altering the electronic delocalization of the hybrid structure.

To explore interactions with biomolecules, alanine was selected as a model amino acid, representing common protein functional groups. Two binding orientations were investigated: via the carboxylic acid (COOH) group and via the amino (NH₂) group. Upon binding, the TDM values decreased notably to 2.81 Debye (COOH-bound) and 2.77 Debye (NH₂-bound), while ΔE increased to 2.20 eV and 2.08 eV, respectively. These changes suggest that alanine binding reduces the overall polarity and reactivity of the composite, likely due to stabilization through hydrogen bonding, coordination interactions, and charge redistribution. The slightly higher ΔE observed for the COOH-bound system implies a marginally more stabilized and less reactive configuration compared to the NH₂-bound counterpart.

**Table 1:** Calculated total dipole moments (TDM, Debye) and HOMO–LUMO band gap energies (ΔE, eV) at the B3LYP/LANL2DZ level of theory.

Structure	TDM (Debye)	ΔE (eV)
Alanine	1.89	5.46
GrO	1.99	2.70
PPy	2.19	4.26
GrO/PPy	5.71	1.36
GrO/PPy/ZnO	5.62	1.64
GrO/PPy/ZnO/COOH	2.81	2.20
GrO/PPy/ZnO/NH₂	2.77	2.08

### Quantum theory of atoms in molecules (QTAIM)

The Quantum Theory of Atoms in Molecules (QTAIM) provides a rigorous quantum mechanical framework for analyzing the topology of electron density within molecular systems. By identifying critical points-bond critical points (BCPs), ring critical points (RCPs), and nuclear critical points (NCPs)-along with the bond paths connecting them, QTAIM enables detailed characterization of both covalent and non-covalent interactions, including hydrogen bonding, van der Waals forces, and coordination bonds. This method offers valuable insight into molecular stability, reactivity, and electronic delocalization phenomena that govern intermolecular interactions^37–39^.

**Fig. 3:**
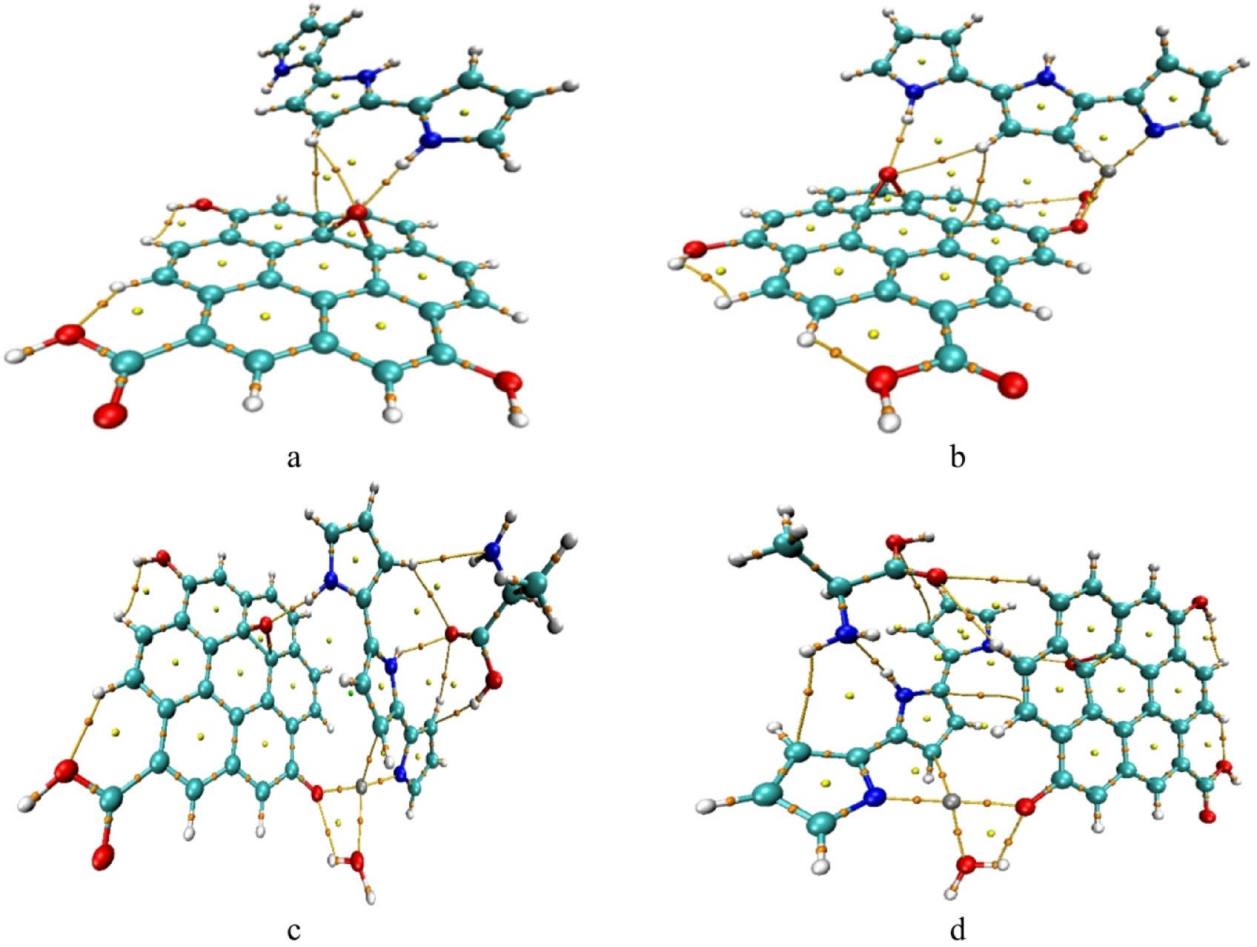
QTAIM calculated for the studied structures whereas; a- GrO/PPy, b- GrO/PPy/ZnO, c- GrO/PPy/ZnO/NH_2_, and d- GrO/PPy/ZnO/COOH.

For the unmodified GrO/PPy composite (Fig. 3a), QTAIM descriptors reveal strong covalent C–C and C–N bonds (ρ(r) > 0.25 e/Å³, H(r) < 0) and moderate closed-shell hydrogen bonds at O–H···O contacts, indicative of a stable covalent framework reinforced by interfacial hydrogen bonding.

Upon ZnO incorporation (Fig. 3b), new Zn–O coordination bonds (ρ(r) ≈ 0.05–0.07 e/Å³, ∇²ρ > 0) emerge in addition to the existing covalent and hydrogen-bond networks. These coordination interactions potentially enhance electronic coupling and increase surface reactivity.

The QTAIM parameters for the alanine-functionalized composites (Tables 1 and 2) reveal distinct binding characteristics for the two functional groups. In the NH₂-bound system (Fig. 3c; Table 1), multiple Zn–N and Zn–O coordination bonds coexist with strong hydrogen bonds (ρ(r) ≈ 0.022–0.025 e/Å³, positive ∇²ρ), forming a flexible interaction network. Elevated ellipticity values for certain BCPs suggest adaptable binding geometries, which may favor versatile molecular recognition. In contrast, the COOH-bound system (Fig. 3d; Table 2) contains fewer but stronger Zn–O coordination and O–H···O hydrogen bonds, resulting in a more localized and rigid binding configuration. This rigidity may improve selectivity but could reduce the diversity of possible interactions. The QTAIM topological parameters for the GrO/PPy/ZnO/COOH composite were listed in Table 3. The table lists the bond critical point (BCP) indices, bond types, electron density ρ(r), kinetic energy density G(r), potential energy density V(r), total energy density H(r), Laplacian of electron density ∇²ρ(r), and ellipticity (ε). Interaction types are classified according to QTAIM criteria into covalent, coordination, hydrogen-bond, van der Waals, and weak covalent interactions.

**Table 2:** QTAIM topological parameters for the GrO/PPy/ZnO/NH_2_ composite.

CP	Bond	ρ(*r*)	G(*r*)	V(*r*)	H(*r*)	∇²ρ(*r*)	ε	Interaction Type
87	H68-O67	0.0249	0.0258	-0.0226	0.0032	0.1160	0.685	Strong hydrogen bond
89	C9-C10	0.2873	0.1026	-0.3893	-0.2868	-0.7368	0.221	Covalent
**90**	**O69-Zn70**	**0.0545**	**0.0689**	**-0.0774**	**-0.0085**	**0.2990**	**0.079**	**Coordination Bond**
91	C9-N13	0.2786	0.1777	-0.4884	-0.3107	-0.5321	0.132	Covalent
92	H16-C10	0.2643	0.0448	-0.3004	-0.2557	-0.8437	0.018	Covalent
**93**	**N13-Zn70**	**0.0884**	**0.0982**	**-0.1195**	**-0.0213**	**0.4221**	**0.078**	**Coordination Bond**
**94**	**O67-Zn70**	**0.0812**	**0.1073**	**-0.1210**	**-0.0137**	**0.4902**	**0.053**	**Coordination Bond**
95	H59-C57	0.2627	0.0453	-0.2970	-0.2516	-0.8251	0.016	Covalent
97	C10-C11	0.2700	0.0891	-0.3423	-0.2531	-0.6558	0.156	Covalent
98	O67-C54	0.2735	0.2101	-0.5367	-0.3266	-0.4657	0.027	Covalent
99	N13-C12	0.2766	0.1512	-0.4382	-0.2870	-0.5431	0.163	Covalent
100	C57-C54	0.2945	0.1018	-0.3999	-0.2981	-0.7853	0.194	Covalent
101	H58-C56	0.2686	0.0400	-0.3030	-0.2630	-0.8923	0.002	Covalent
**103**	**Zn70-C2**	**0.0321**	**0.0233**	**-0.0316**	**-0.0083**	**0.0685**	**0.181**	**Coordination Bond**
104	C11-C12	0.2817	0.1020	-0.3798	-0.2777	-0.7028	0.210	Covalent
105	C57-C52	0.2776	0.0872	-0.3529	-0.2657	-0.7138	0.130	Covalent
106	C54-C48	0.2693	0.0808	-0.3270	-0.2462	-0.6619	0.123	Covalent
107	C11-H17	0.2645	0.0447	-0.3013	-0.2566	-0.8477	0.024	Covalent
108	C56-C52	0.2803	0.0854	-0.3549	-0.2695	-0.7366	0.095	Covalent
109	H49-C43	0.2656	0.0442	-0.3008	-0.2566	-0.8497	0.008	Covalent
110	O61-C60	0.3629	0.3888	-0.9247	-0.5359	-0.5886	0.053	Covalent
111	C12-C1	0.2609	0.0738	-0.3070	-0.2332	-0.6376	0.114	Covalent
112	H7-C2	0.2549	0.0491	-0.2892	-0.2402	-0.7644	0.044	Covalent
**113**	**C11-H82**	**0.0140**	**0.0097**	**-0.0091**	**0.0007**	**0.0417**	**0.387**	**van der Waals**
115	C56-C53	0.2956	0.1012	-0.4022	-0.3010	-0.7994	0.174	Covalent
116	C48-C43	0.2666	0.0793	-0.3234	-0.2441	-0.6596	0.091	Covalent
117	C2-C1	0.2755	0.0958	-0.3598	-0.2640	-0.6726	0.203	Covalent
118	C60-C53	0.2465	0.0730	-0.2806	-0.2076	-0.5385	0.087	Covalent
119	C52-C46	0.2758	0.0844	-0.3447	-0.2603	-0.7037	0.116	Covalent
120	C60-O62	0.2583	0.1771	-0.4627	-0.2857	-0.4343	0.002	Covalent
121	H82-O81	0.3098	0.0582	-0.4641	-0.4058	-1.3905	0.017	Covalent
122	C48-C42	0.2828	0.0920	-0.3672	-0.2752	-0.7327	0.155	Covalent
124	H63-O62	0.3201	0.0605	-0.4799	-0.4194	-1.4355	0.020	Covalent
126	C43-C39	0.3002	0.1058	-0.4177	-0.3119	-0.8241	0.187	Covalent
127	C1-N5	0.2842	0.1957	-0.5251	-0.3295	-0.5353	0.130	Covalent
**129**	**H17-O83**	**0.0094**	**0.0073**	**-0.0054**	**0.0019**	**0.0365**	**0.098**	**Weak hydrogen bond**
131	C2-C3	0.2637	0.0846	-0.3256	-0.2410	-0.6254	0.144	Covalent
132	C46-C42	0.2827	0.0910	-0.3653	-0.2743	-0.7333	0.148	Covalent
133	C53-C47	0.2618	0.0771	-0.3119	-0.2347	-0.6304	0.116	Covalent
136	C39-H44	0.2644	0.0430	-0.2971	-0.2541	-0.8444	0.004	Covalent
137	C46-C41	0.2625	0.0763	-0.3115	-0.2352	-0.6357	0.096	Covalent
139	C42-C36	0.2588	0.0715	-0.2975	-0.2260	-0.6180	0.059	Covalent
140	O81-C71	0.2695	0.1981	-0.5107	-0.3126	-0.4579	0.029	Covalent
141	C3-H8	0.2661	0.0434	-0.3030	-0.2596	-0.8645	0.019	Covalent
142	C47-C41	0.2696	0.0822	-0.3306	-0.2485	-0.6652	0.124	Covalent
143	C39-C33	0.2724	0.0829	-0.3383	-0.2554	-0.6900	0.105	Covalent
144	N5-H6	0.3124	0.0399	-0.4400	-0.4001	-1.4410	0.030	Covalent
**145**	**O62-H55**	**0.0230**	**0.0240**	**-0.0210**	**0.0030**	**0.1083**	**0.037**	**Strong hydrogen bond**
146	C3-C4	0.2924	0.1080	-0.4055	-0.2976	-0.7585	0.241	Covalent
**147**	**H6-O83**	**0.0122**	**0.0116**	**-0.0086**	**0.0030**	**0.0584**	**0.261**	**Moderate hydrogen bond**
148	N5-C4	0.2655	0.1767	-0.4702	-0.2934	-0.4669	0.127	Covalent
150	C47-C50	0.2749	0.0855	-0.3460	-0.2605	-0.6997	0.108	Covalent
151	C71-O83	0.3601	0.4002	-0.9337	-0.5335	-0.5333	0.028	Covalent
152	C36-C33	0.2560	0.0717	-0.2934	-0.2217	-0.6002	0.062	Covalent
153	C41-C35	0.2783	0.0882	-0.3539	-0.2657	-0.7099	0.143	Covalent
154	H55-C50	0.2737	0.0400	-0.3143	-0.2743	-0.9372	0.005	Covalent
156	C71-C72	0.2350	0.0665	-0.2531	-0.1866	-0.4806	0.072	Covalent
157	C36-O64	0.1813	0.1172	-0.2436	-0.1264	-0.0368	0.311	Weak Covalent
158	C36-C31	0.2157	0.0684	-0.2298	-0.1614	-0.3723	0.071	Covalent
160	H79-C75	0.2588	0.0465	-0.2906	-0.2441	-0.7903	0.017	Covalent
161	C33-C30	0.2923	0.0987	-0.3935	-0.2948	-0.7847	0.164	Covalent
162	H74-C72	0.2539	0.0442	-0.2757	-0.2314	-0.7489	0.022	Covalent
163	C4-C18	0.2591	0.0705	-0.2996	-0.2292	-0.6350	0.103	Covalent
165	C50-C45	0.2969	0.1027	-0.4079	-0.3052	-0.8096	0.166	Covalent
166	C35-C31	0.2547	0.0693	-0.2875	-0.2181	-0.5952	0.060	Covalent
167	C72-C75	0.2203	0.0575	-0.2215	-0.1640	-0.4261	0.020	Covalent
169	C31-O64	0.1874	0.1188	-0.2547	-0.1359	-0.0685	0.272	Weak Covalent
170	C35-C40	0.2837	0.0927	-0.3697	-0.2769	-0.7373	0.161	Covalent
171	C30-H34	0.2646	0.0425	-0.2969	-0.2544	-0.8477	0.002	Covalent
**172**	**O83-H24**	**0.0028**	**0.0019**	**-0.0012**	**0.0007**	**0.0106**	**1.131**	**Very weak hydrogen bond**
173	C75-H78	0.2584	0.0464	-0.2897	-0.2433	-0.7874	0.016	Covalent
175	C72-N73	0.2471	0.1017	-0.3216	-0.2199	-0.4728	0.087	Covalent
**176**	**O64-H23**	**0.0225**	**0.0213**	**-0.0195**	**0.0018**	**0.0923**	**0.049**	**Strong hydrogen bond**
177	C45-C40	0.2769	0.0875	-0.3523	-0.2647	-0.7088	0.123	Covalent
178	C31-C29	0.2537	0.0694	-0.2861	-0.2167	-0.5891	0.057	Covalent
179	C75-H80	0.2548	0.0488	-0.2856	-0.2368	-0.7519	0.018	Covalent
180	C30-C28	0.2774	0.0870	-0.3527	-0.2657	-0.7148	0.119	Covalent
181	C18-N22	0.2719	0.1823	-0.4864	-0.3041	-0.4870	0.186	Covalent
182	H23-N22	0.3087	0.0405	-0.4347	-0.3942	-1.4145	0.030	Covalent
183	C45-H51	0.2614	0.0470	-0.2955	-0.2485	-0.8057	0.007	Covalent
185	C18-C19	0.2864	0.1052	-0.3919	-0.2867	-0.7261	0.228	Covalent
186	H76-N73	0.3139	0.0491	-0.4451	-0.3960	-1.3876	0.051	Covalent
187	C29-C28	0.2912	0.0987	-0.3919	-0.2932	-0.7779	0.167	Covalent
188	C40-C37	0.2618	0.0766	-0.3103	-0.2337	-0.6281	0.109	Covalent
**189**	**N73-H24**	**0.0044**	**0.0028**	**-0.0018**	**0.0009**	**0.0148**	**0.067**	**Weak hydrogen bond**
190	H24-C19	0.2654	0.0423	-0.3007	-0.2584	-0.8641	0.019	**Covalent**
191	C29-C32	0.2750	0.0850	-0.3458	-0.2608	-0.7035	0.121	**Covalent**
193	C28-H27	0.2643	0.0435	-0.2974	-0.2539	-0.8419	0.008	**Covalent**
195	N73-H77	0.3153	0.0512	-0.4468	-0.3956	-1.3778	0.052	**Covalent**
**196**	**H51-H66**	**0.0128**	**0.0112**	**-0.0089**	**0.0023**	**0.0542**	**0.556**	**van der Waals**
197	C37-C32	0.3027	0.1130	-0.4323	-0.3193	-0.8250	0.221	**Covalent**
198	N22-C21	0.2742	0.2033	-0.5223	-0.3190	-0.4629	0.137	**Covalent**
199	C19-C20	0.2696	0.0887	-0.3411	-0.2524	-0.6546	0.152	**Covalent**
200	C37-O65	0.2504	0.1943	-0.4824	-0.2881	-0.3754	0.013	**Covalent**
201	C32-H38	0.2647	0.0429	-0.2993	-0.2564	-0.8536	0.016	**Covalent**
202	C21-C20	0.2899	0.1054	-0.3986	-0.2932	-0.7510	0.231	**Covalent**
203	H66-O65	0.3285	0.0639	-0.4954	-0.4315	-1.4705	0.022	**Covalent**
204	C21-H26	0.2681	0.0424	-0.3051	-0.2627	-0.8812	0.034	**Covalent**
205	C20-H25	0.2646	0.0451	-0.3014	-0.2564	-0.8451	0.019	**Covalent**

The table lists the bond critical point (BCP) indices, bond types, electron density ρ(r), kinetic energy density G(r), potential energy density V(r), total energy density H(r), laplacian of electron density ∇²ρ(r), and ellipticity (ε). Interaction types are classified according to QTAIM criteria into covalent, coordination, hydrogen-bond, Van der waals, and weak covalent interactions.

**Table 3:** QTAIM topological parameters for the GrO/PPy/ZnO/COOH composite.

CP	Bond	ρ(*r*)	G(*r*)	V(*r*)	H(*r*)	∇²ρ	ε	Interaction Type
84	H25–C20	0.2640	0.0455	-0.3008	-0.2553	-0.8392	0.0201	Covalent
85	H83–O82	0.3192	0.0586	-0.4776	-0.4190	-1.4415	0.0181	Covalent
86	H26–C21	0.2685	0.0420	-0.3055	-0.2635	-0.8860	0.0336	Covalent
87	C20–C21	0.2898	0.1050	-0.3976	-0.2926	-0.7504	0.2329	Covalent
**88**	**O82–C19**	**0.0045**	**0.0028**	**-0.0021**	**0.0008**	**0.0144**	**3.6595**	**Van der Waals**
89	C20–C19	0.2688	0.0885	-0.3394	-0.2509	-0.6498	0.1568	Covalent
90	O82–C77	0.2681	0.1959	-0.5051	-0.3092	-0.4534	0.0323	Covalent
91	H79–C76	0.2570	0.0465	-0.2875	-0.2409	-0.7777	0.0149	Covalent
92	H38–C32	0.2645	0.0432	-0.2991	-0.2559	-0.8511	0.0157	Covalent
93	H27–C28	0.2638	0.0440	-0.2968	-0.2528	-0.8354	0.0075	Covalent
94	C21–N22	0.2747	0.2015	-0.5205	-0.3190	-0.4702	0.1349	Covalent
95	H24–C19	0.2639	0.0458	-0.3007	-0.2549	-0.8365	0.0226	Covalent
97	C77–O81	0.3647	0.4139	-0.9589	-0.5450	-0.5244	0.0356	Covalent
98	H75–C74	0.2657	0.0417	-0.2956	-0.2539	-0.8491	0.0197	Covalent
99	C77–C74	0.2361	0.0657	-0.2531	-0.1874	-0.4871	0.0612	Covalent
100	C76–C74	0.2171	0.0571	-0.2154	-0.1582	-0.4045	0.0206	Covalent
101	O65–H66	0.3286	0.0640	-0.4955	-0.4315	-1.4700	0.0218	Covalent
102	C76–H80	0.2556	0.0480	-0.2863	-0.2382	-0.7608	0.0157	Covalent
103	C76–H78	0.2561	0.0475	-0.2864	-0.2388	-0.7653	0.0159	Covalent
104	C32–C37	0.3027	0.1131	-0.4325	-0.3194	-0.8250	0.2218	Covalent
105	C19–C18	0.2875	0.1047	-0.3925	-0.2878	-0.7326	0.2364	Covalent
106	C32–C29	0.2747	0.0848	-0.3452	-0.2604	-0.7023	0.1212	Covalent
107	O65–C37	0.2498	0.1936	-0.4807	-0.2871	-0.3740	0.0128	Covalent
108	C28–C29	0.2912	0.0988	-0.3919	-0.2931	-0.7772	0.1668	Covalent
**109**	**O81–H34**	**0.0070**	**0.0060**	**-0.0039**	**0.0021**	**0.0323**	**0.0511**	**Weak hydrogen bond**
110	C28–C30	0.2773	0.0868	-0.3521	-0.2653	-0.7142	0.1134	Covalent
111	H34–C30	0.2670	0.0404	-0.3000	-0.2596	-0.8770	0.0015	Covalent
113	N22–C18	0.2728	0.1835	-0.4897	-0.3062	-0.4907	0.1728	Covalent
114	C74–N72	0.2389	0.0966	-0.3015	-0.2049	-0.4334	0.0618	Covalent
115	N22–H23	0.3091	0.0402	-0.4350	-0.3948	-1.4185	0.0301	Covalent
**116**	**H66–H51**	**0.0128**	**0.0112**	**-0.0089**	**0.0023**	**0.0542**	**0.5542**	**van der Waals**
118	C29–C31	0.2536	0.0694	-0.2859	-0.2166	-0.5888	0.0568	Covalent
120	C37–C40	0.2618	0.0767	-0.3104	-0.2337	-0.6280	0.1094	Covalent
**122**	**H23–O64**	**0.0213**	**0.0202**	**-0.0182**	**0.0019**	**0.0884**	**0.0519**	**Strong hydrogen bond**
125	C30–C33	0.2924	0.0986	-0.3935	-0.2949	-0.7853	0.1591	Covalent
126	C18–C4	0.2588	0.0696	-0.2976	-0.2280	-0.6339	0.1025	Covalent
**127**	**O81–H44**	**0.0062**	**0.0052**	**-0.0033**	**0.0018**	**0.0281**	**0.0983**	**Weak hydrogen bond**
128	O64–C31	0.1863	0.1181	-0.2519	-0.1338	-0.0631	0.2769	Covalent
**130**	**N72–H6**	**0.0389**	**0.0295**	**-0.0329**	**-0.0034**	**0.1044**	**0.0086**	**Strong hydrogen Bond**
131	N72–H73	0.3101	0.0491	-0.4351	-0.3860	-1.3476	0.0418	Covalent
132	N72–H71	0.3111	0.0494	-0.4368	-0.3874	-1.3518	0.0414	Covalent
133	C40–C45	0.2768	0.0875	-0.3521	-0.2646	-0.7084	0.1235	Covalent
134	C31–C35	0.2547	0.0693	-0.2873	-0.2181	-0.5951	0.0596	Covalent
135	C40–C35	0.2838	0.0927	-0.3697	-0.2770	-0.7374	0.1614	Covalent
136	H51–C45	0.2614	0.0472	-0.2955	-0.2483	-0.8048	0.0068	Covalent
137	O64–C36	0.1789	0.1162	-0.2386	-0.1224	-0.0250	0.3273	Covalent
138	C31–C36	0.2174	0.0685	-0.2325	-0.1640	-0.3821	0.0662	Covalent
139	H6–N5	0.2881	0.0427	-0.4026	-0.3599	-1.2687	0.0282	Covalent
141	C33–C36	0.2565	0.0722	-0.2950	-0.2228	-0.6026	0.0615	Covalent
142	C4–N5	0.2677	0.1687	-0.4600	-0.2913	-0.4905	0.1320	Covalent
143	C33–C39	0.2728	0.0830	-0.3390	-0.2560	-0.6920	0.1014	Covalent
144	H44–C39	0.2665	0.0415	-0.3000	-0.2586	-0.8684	0.0027	Covalent
145	C4–C3	0.2924	0.1079	-0.4053	-0.2974	-0.7580	0.2410	Covalent
**146**	**C4–C43**	**0.0029**	**0.0016**	**-0.0010**	**0.0006**	**0.0086**	**3.3023**	**Van der Waals**
**147**	**H71–C11**	**0.0048**	**0.0031**	**-0.0020**	**0.0011**	**0.0170**	**0.4124**	**Van der Waals**
149	C45–C50	0.2968	0.1027	-0.4077	-0.3050	-0.8090	0.1665	Covalent
151	C35–C41	0.2782	0.0881	-0.3536	-0.2654	-0.7091	0.1432	Covalent
153	H8–C3	0.2657	0.0436	-0.3024	-0.2588	-0.8606	0.0184	Covalent
155	C36–C42	0.2591	0.0717	-0.2982	-0.2265	-0.6195	0.0590	Covalent
157	N5–C1	0.2869	0.1916	-0.5222	-0.3306	-0.5558	0.1245	Covalent
158	C39–C43	0.3001	0.1057	-0.4174	-0.3117	-0.8240	0.1836	Covalent
159	C3–C2	0.2627	0.0839	-0.3228	-0.2389	-0.6201	0.1386	Covalent
161	H17–C11	0.2622	0.0465	-0.2983	-0.2518	-0.8210	0.0220	Covalent
162	C50–H55	0.2736	0.0401	-0.3142	-0.2741	-0.9363	0.0046	Covalent
163	C50–C47	0.2749	0.0855	-0.3460	-0.2604	-0.6996	0.1085	Covalent
164	C41–C47	0.2697	0.0822	-0.3307	-0.2486	-0.6655	0.1241	Covalent
165	C1–C2	0.2752	0.0951	-0.3580	-0.2628	-0.6707	0.1964	Covalent
166	C41–C46	0.2626	0.0763	-0.3118	-0.2354	-0.6363	0.0958	Covalent
167	C42–C46	0.2828	0.0910	-0.3656	-0.2745	-0.7340	0.1478	Covalent
168	C1–C12	0.2603	0.0728	-0.3047	-0.2318	-0.6360	0.1040	Covalent
169	C42–C48	0.2828	0.0920	-0.3672	-0.2751	-0.7324	0.1554	Covalent
170	C43–C48	0.2665	0.0794	-0.3234	-0.2441	-0.6589	0.0918	Covalent
171	C43–H49	0.2650	0.0448	-0.3002	-0.2554	-0.8424	0.0087	Covalent
172	C11–C12	0.2851	0.1038	-0.3879	-0.2840	-0.7207	0.2258	Covalent
173	C2–H7	0.2563	0.0480	-0.2904	-0.2425	-0.7780	0.0403	Covalent
**174**	**H55–O62**	**0.0230**	**0.0240**	**-0.0210**	**0.0030**	**0.1082**	**0.0362**	**Strong hydrogen Bond**
175	C11–C10	0.2693	0.0893	-0.3414	-0.2521	-0.6513	0.1637	Covalent
176	C47–C53	0.2617	0.0771	-0.3117	-0.2346	-0.6299	0.1160	Covalent
179	C46–C52	0.2758	0.0844	-0.3446	-0.2603	-0.7036	0.1155	Covalent
181	C12–N13	0.2753	0.1520	-0.4374	-0.2854	-0.5339	0.1692	Covalent
**183**	**C2–Zn70**	**0.0330**	**0.0235**	**-0.0323**	**-0.0088**	**0.0676**	**0.1786**	**Coordinate Covalent**
184	C48–C54	0.2691	0.0806	-0.3263	-0.2457	-0.6606	0.1215	Covalent
186	C10–H16	0.2635	0.0455	-0.2996	-0.2541	-0.8346	0.0192	Covalent
187	C53–C56	0.2953	0.1011	-0.4016	-0.3006	-0.7981	0.1736	Covalent
188	C10–C9	0.2858	0.1016	-0.3854	-0.2838	-0.7289	0.2244	Covalent
**189**	**N13–Zn70**	**0.0873**	**0.0967**	**-0.1177**	**-0.0210**	**0.4148**	**0.0788**	**Coordinate Covalent**
190	C53–C60	0.2467	0.0733	-0.2814	-0.2081	-0.5395	0.0882	Covalent
191	C52–C56	0.2803	0.0854	-0.3550	-0.2696	-0.7367	0.0955	Covalent
192	C52–C57	0.2776	0.0873	-0.3529	-0.2657	-0.7136	0.1300	Covalent
193	C54–O67	0.2746	0.2123	-0.5413	-0.3291	-0.4673	0.0264	Covalent
194	C54–C57	0.2943	0.1015	-0.3991	-0.2975	-0.7840	0.1932	Covalent
195	N13–C9	0.2790	0.1796	-0.4917	-0.3121	-0.5302	0.1400	Covalent
196	O62–C60	0.2579	0.1766	-0.4615	-0.2849	-0.4333	0.0023	Covalent
197	O62–H63	0.3202	0.0606	-0.4801	-0.4195	-1.4353	0.0196	Covalent
**198**	**Zn70–O67**	**0.0798**	**0.1053**	**-0.1187**	**-0.0134**	**0.4807**	**0.0506**	**Coordinate Covalent**
199	C60–O61	0.3627	0.3881	-0.9235	-0.5354	-0.5893	0.0527	Covalent
200	C56–H58	0.2686	0.0400	-0.3029	-0.2630	-0.8918	0.0016	Covalent
**201**	**Zn70–O69**	**0.0539**	**0.0679**	**-0.0764**	**-0.0085**	**0.2938**	**0.0765**	**Coordinate Covalent**
202	C9–H15	0.2660	0.0441	-0.3019	-0.2578	-0.8549	0.0330	Covalent
203	C57–H59	0.2627	0.0454	-0.2970	-0.2516	-0.8251	0.0166	Covalent
**205**	**O67–H68**	**0.0258**	**0.0266**	**-0.0236**	**0.0030**	**0.1184**	**0.5860**	**Strong hydrogen Bond**
206	O69–H68	0.3072	0.0567	-0.4635	-0.4069	-1.4008	0.0209	Covalent
207	O69–H14	0.3270	0.0600	-0.4934	-0.4333	-1.4933	0.0205	Covalent

The table lists the bond critical point (BCP) indices, bond types, electron density ρ(r), kinetic energy density G(r), potential energy density V(r), total energy density H(r), laplacian of electron density ∇²ρ(r), and ellipticity (ε). Interaction types are classified according to QTAIM criteria into covalent, coordination, hydrogen-bond, Van der waals, and weak covalent interactions.

### Molecular electrostatic potential (MESP)

**Fig. 4:**
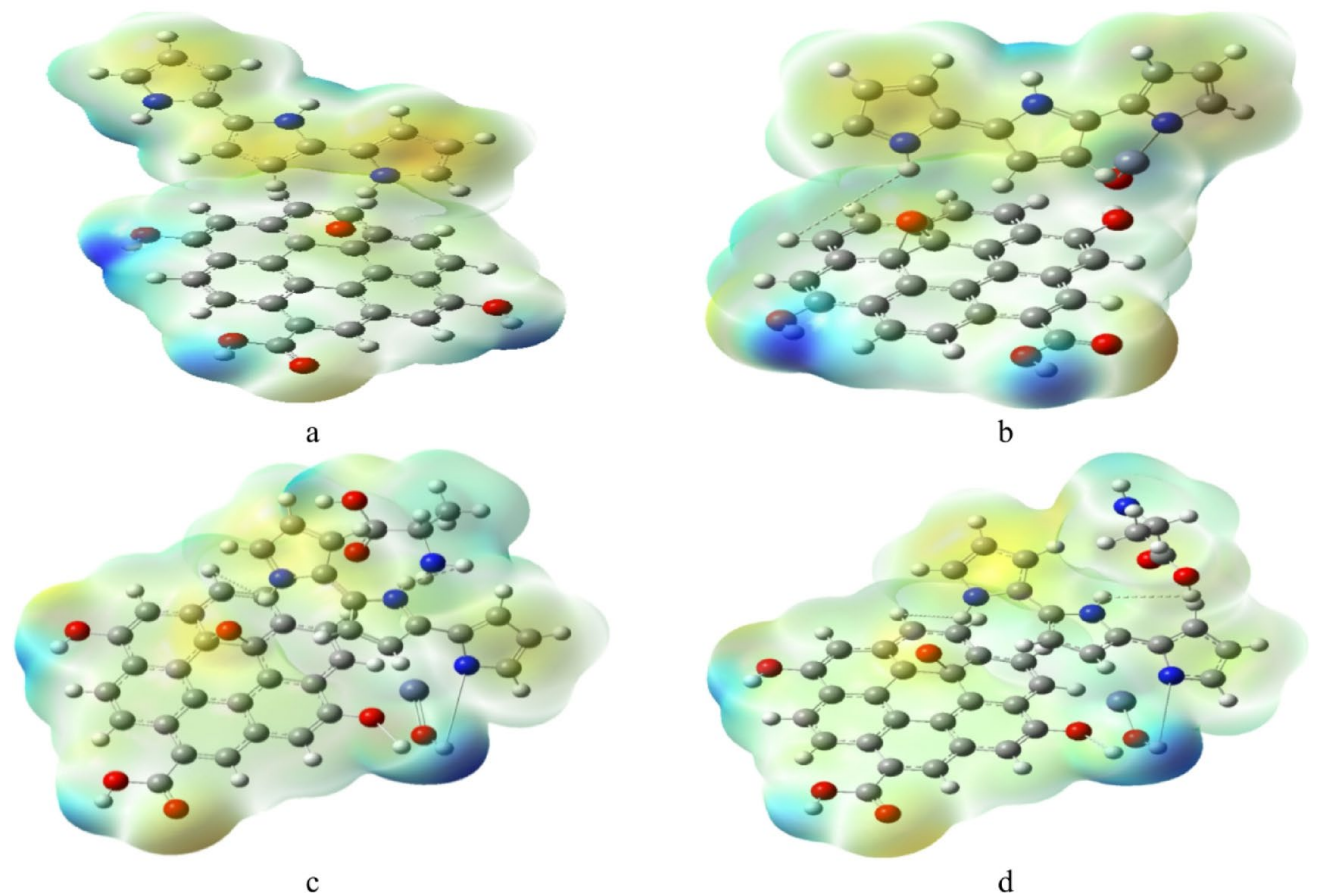
MESP calculated for the studied structures whereas; a- GrO/PPy, b- GrO/PPy/ZnO, c- GrO/PPy/ZnO/NH_2_, and d- GrO/PPy/ZnO/COOH.

The molecular electrostatic potential (MESP) maps of the studied systems (Fig. 4) provide insight into surface charge distribution and the locations most favorable for alanine interaction.

The unmodified GrO/PPy composite (Fig. 4a) shows a balanced distribution of electron-rich (blue) and electron-deficient (red/yellow) regions, indicating a baseline electrostatic profile with moderate polarity. Incorporation of ZnO (Fig. 4b) increases the heterogeneity of the electrostatic potential, particularly at the interface regions, creating additional polar sites that can serve as potential binding points for alanine.

When alanine is bound through its NH₂ group (Fig. 4c), the composite surface displays an enhanced positive potential near the amine binding site, reflecting stronger hydrogen-bond donor capability toward electron-rich regions of the alanine molecule. In contrast, when alanine is bound via its COOH group (Fig. 4d), the MESP reveals pronounced dipolar features around the carboxylate binding region, suggesting an increased ability to interact with polar or hydrogen-bond-accepting regions on the composite.

These differences indicate that NH₂ binding promotes a more flexible interaction profile, whereas COOH binding results in a more localized, high-polarity binding environment. Such variation in electrostatic surface characteristics may influence the sensitivity and selectivity of GrO–PPy–ZnO toward alanine detection.

### Global reactivity descriptors

Global reactivity descriptors quantitatively describe molecular reactivity and electronic properties, providing key insights into chemical behavior and sensor performance. The ionization potential (I), electron affinity (A), electronic chemical potential (µ), chemical hardness (η), absolute softness (S), and electrophilicity index (ω) were calculated from frontier molecular orbital energies (HOMO and LUMO) according to established equations^40^: I = − E_HOMO_, A = − E_LUMO_, µ = −(I + A)/2, η = (I − A)/2, S = 1/η, and ω = µ²/2η.

Table 4 presents the B3LYP/LANL2DZ-calculated descriptors for pristine GrO, PPy, the GrO/PPy/ZnO composite, and its COOH- and NH₂-functionalized derivatives upon interaction with alanine.

The pristine GrO/PPy/ZnO composite shows an ionization potential of 3.03 eV, which decreases to 2.56 eV for the COOH-functionalized system and 2.67 eV for the NH₂-functionalized variant after alanine binding. This reduction indicates greater ease of electron removal, enhancing electronic accessibility at the sensor surface. Concurrently, electron affinity increases in both functionalized systems, signifying an improved electron-accepting capability and promoting charge transfer processes critical for sensing.

Chemical hardness (η) and absolute softness (S) change modestly after binding, suggesting slight adjustments in structural flexibility without compromising stability. Notably, electrophilicity index (ω) and electronegativity (χ) decrease upon alanine interaction, implying reduced electron-withdrawing tendency, which can paradoxically favor selective recognition by lowering the energy barrier for analyte–sensor interaction.

Overall, the COOH-functionalized composite demonstrates the most favorable combination of low ionization potential and high electron affinity, producing an electronic environment well-suited for selective alanine detection. These correlations between global reactivity descriptors and binding behavior support the potential of functionalized GrO/PPy/ZnO as a high-performance chemical sensor^41^.

**Table 4:** B3LYP/LANL2DZ-calculated global reactivity descriptors for studied structures.

Structure	I (eV)	A (eV)	η (eV)	S (eV⁻¹)	ω (eV)	χ (eV)
Alanine	0.63	6.09	-3.36	-2.73	-0.37	-2.06
**GrO**	2.67	5.38	-4.02	-1.35	-0.74	-5.99
PPy	0.46	4.72	-2.59	-2.13	-0.47	-1.57
GrO/PPy	2.95	4.31	-3.63	-0.68	-1.47	-9.65
GrO/PPy/ZnO	3.03	4.68	-3.86	-0.82	-1.22	-9.04
GrO/PPy/ZnO/COOH	2.56	4.77	-3.66	-1.10	-0.91	-6.10
GrO/PPy/ZnO/NH₂	2.67	4.75	-3.71	-1.04	-0.96	-6.62

### Density of States DOS

**Fig. 5:**
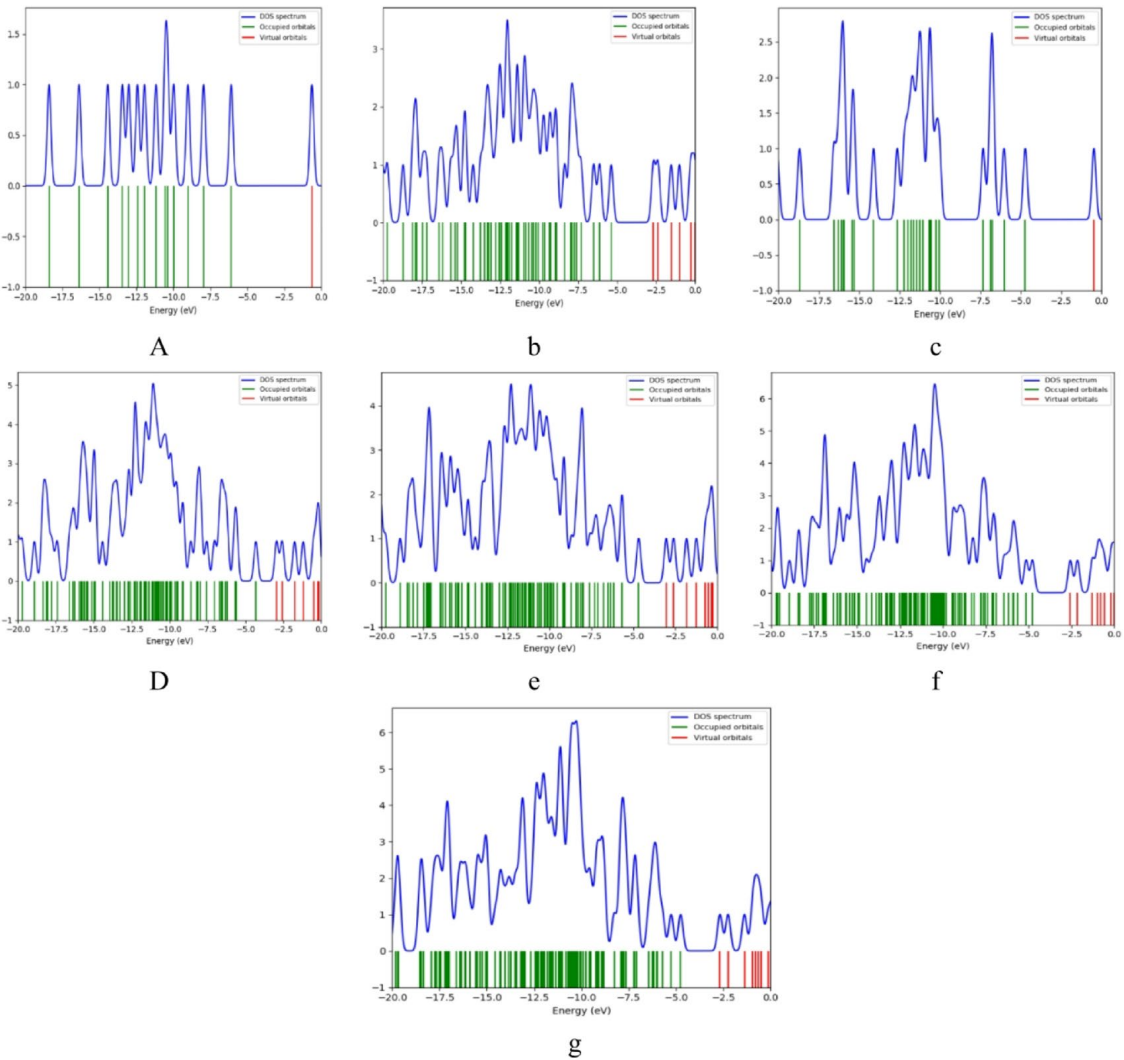
Density of states for the studied structures whereas; a- Alanine, b- Graphene oxide (GrO), c- Polypyrrole (PPy); d- GrO/PPy, e- GrO/PPy/ZnO, f- GrO/PPy/ZnO/COOH, and g- GrO/PPy/ZnO/NH_2_.

The density of states (DOS) is calculated also at B3LYP/LANL2DZ and plotted with the help of Gauss sum program^41^. The DOS describes the number of allowed modes or states per unit energy range. Figure 5 illustrates the DOS for the interactions of GrO with PPy, as well as with ZnO through the COOH and NH_2_ functional groups. As indicated in Fig. 5a and c, both alanine and PPy exhibit the largest energy band gaps. In contrast, GrO demonstrates a considerably smaller energy gap, as shown in Fig. 5b. This phenomenon can be explained by the electronic structure of graphene oxide, which typically features a reduced band gap due to its sp² hybridized carbon atoms and the presence of oxygen-containing functional groups. When GrO interacts with PPy, or when ZnO is involved with GrO/PPy, there is a significant decrease in the energy band gap, as illustrated in Fig. 5d and e, respectively. This reduction is likely a result of the synergistic interaction between ZnO and GrO/PPy, where ZnO may either donate or accept electrons, thereby modifying the electronic characteristics of the composite material. Such interactions lead to a shift in energy levels, which diminishes the band gap and enhances charge transfer capabilities.

The Density of States (DOS) analysis shows changes in the band gap upon interaction. Furthermore, the energy gap also diminishes when interactions occur via the COOH group, as shown in Fig. 5f, and similarly when the NH_2_ group is involved, as depicted in Fig. 5g. These findings indicate that the COOH and NH_2_ functional groups significantly influence the electronic structure and promote electron transfer. The most pronounced alteration in the DOS and the lowest energy gap are observed in the GrO/PPy/ZnO composite, as emphasized in the figures. This observation aligns with the calculated energy gap, suggesting that the combination of GrO, PPy, and ZnO leads to an optimized electronic structure, thereby enhancing the overall electronic properties of the material. This composite is particularly advantageous for applications requiring efficient charge transport, such as in sensors, energy storage systems, or catalysis.

### Non-Covalent interactions (NCI) and reduced density gradient (RDG) analysis

**Fig. 6:**
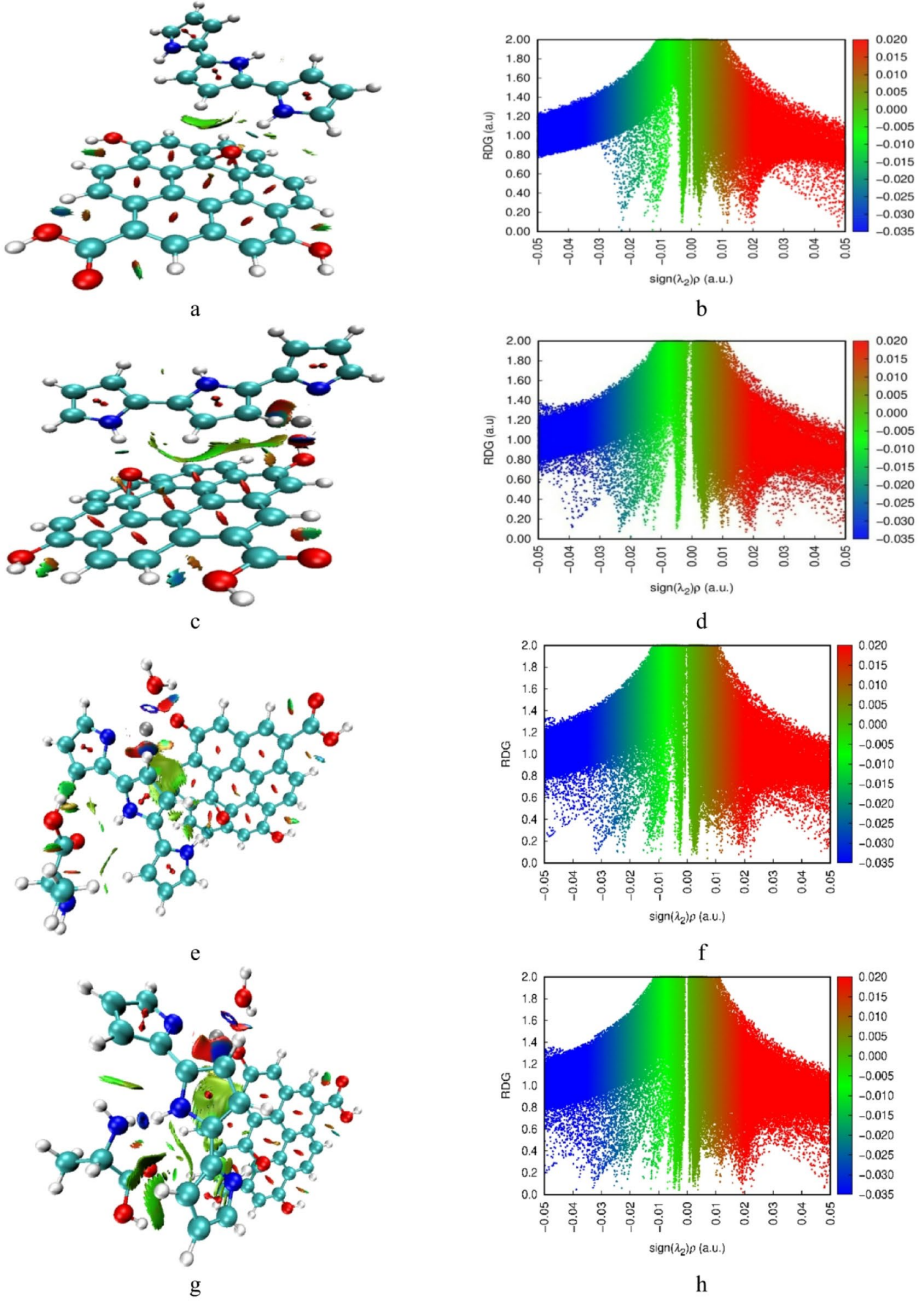
NCI (a, c, e, g) and RDG (b, d, f, h) calculated for the studied structures: a/b - GrO/PPy, c/d - GrO/PPy/ZnO, e/f - GrO/PPy/ZnO/NH_2_, and g/h - GrO/PPy/ZnO/COOH.

Non-covalent interaction (NCI) and reduced density gradient (RDG) analyses were performed to systematically investigate weak intermolecular interactions within the studied nanocomposite systems. These complementary computational methods, visualized through distinct color-coded isosurfaces^42^, provide comprehensive insights into hydrogen bonding networks, van der Waals forces, and steric repulsion effects. The NCI visualization scheme employs a standardized color mapping where red isosurfaces indicate strong repulsive interactions, blue regions represent strong attractive interactions, and green areas correspond to weaker interactions such as van der Waals or dispersion forces. Non-covalent interaction (NCI) and reduced density gradient (RDG) analyses complement QTAIM by visualizing weak interaction domains via color-coded isosurfaces. In the pristine GrO–PPy composite (Fig. 6a, b), extended green isosurfaces dominate the interfacial regions, consistent with π–π stacking and van der Waals dispersion between the polypyrrole and graphene oxide layers. Localized blue areas coincide with hydrogen-bond sites, reinforcing QTAIM findings.

ZnO incorporation (Fig. 6c, d) introduces pronounced blue domains near Zn–O coordination points, signifying strong attractive forces, and a wider spread of green regions along the GrO–ZnO and PPy–ZnO boundaries, indicative of enhanced dispersion stabilization. This expansion of interaction domains implies a more heterogeneous and adsorption-friendly surface.

For the alanine-bound systems, the NH₂-functionalized composite (Fig. 6e, f) shows a broad distribution of green zones interspersed with blue coordination sites, pointing to synergistic effects between dispersion forces and metal–ligand coordination. The COOH-bound composite (Fig. 6g, h) displays more concentrated blue regions, corresponding to fewer but stronger hydrogen bonds and coordination interactions. This spatial distribution supports the QTAIM observation of a more rigid binding environment, which may be beneficial for selective molecular recognition.

## Conclusion

This computational investigation provides a comprehensive understanding of the electronic and interactional characteristics of a graphene oxide–polypyrrole–zinc oxide (GrO/PPy/ZnO) nanocomposite and its NH₂- and COOH-functionalized derivatives for alanine sensing. The pristine composite exhibits an ionization potential (I) of 3.03 eV and an electron affinity (A) of 4.68 eV, supporting balanced electron-donating and electron-accepting abilities. Upon alanine binding, COOH functionalization yields the most pronounced changes, reducing I from 3.03 eV to 2.56 eV and increasing A from 4.68 eV to 4.77 eV, creating an electronic environment highly favorable for charge-transfer-driven sensing. NH₂ functionalization also enhances reactivity, lowering I to 2.67 eV and raising A to 4.75 eV, but with a smaller effect than COOH. Variations in chemical hardness (η) remain modest (from − 3.86 eV in the pristine composite to − 3.66 eV for COOH and − 3.71 eV for NH₂), indicating that reactivity improvements occur without significant loss of structural stability. Absolute softness (S) increases slightly upon functionalization (from − 0.82 eV⁻¹ to − 1.10 eV⁻¹ for COOH and − 1.04 eV⁻¹ for NH₂), reflecting greater flexibility in electronic response. The electrophilicity index (ω) decreases from − 1.22 eV in the pristine system to − 0.91 eV for COOH and − 0.96 eV for NH₂, suggesting a reduced electron-withdrawing tendency that can improve selectivity by favoring energetically accessible target–sensor interactions. Complementary QTAIM, MESP, NCI, and RDG analyses confirm the formation of stable non-covalent interactions and favorable electrostatic potential distributions, with alanine engaging the composite through both NH₂ and COOH binding sites. Collectively, the results identify COOH-functionalized GrO/PPy/ZnO as the most promising candidate for high-performance alanine detection, offering the optimal balance of low ionization potential, high electron affinity, enhanced softness, and maintained stability, thus establishing a robust theoretical foundation for future experimental sensor development.

## Data Availability

The data will be available upon request. Contact Medhat A. Ibrahim, Email: [ma.khalek@nrc.sci.eg]

## Data availability

The data will be available upon request. Contact Medhat A. Ibrahim, Email: ma.khalek@nrc.sci.eg.
